# Cerebrovascular dynamics after pediatric traumatic brain injury

**DOI:** 10.3389/fphys.2023.1093330

**Published:** 2023-02-17

**Authors:** Damla Hanalioglu, Brian T. Burrows, P. David Adelson, Brian Appavu

**Affiliations:** ^1^ Department of Neurosciences, Barrow Neurological Institute at Phoenix Children’s Hospital, Phoenix, AZ, United States; ^2^ Department of Child Health, University of Arizona College of Medicine—Phoenix, Phoenix, AZ, United States

**Keywords:** traumatic brain injury, transcranial Doppler ultrasound, neurocritical care, multimodality monitoring, critical closing pressure, diastolic closing margin

## Abstract

**Objective:** We aimed to investigate model-based indices of cerebrovascular dynamics after pediatric traumatic brain injury (TBI) using transcranial Doppler ultrasound (TCD) integrated into multimodality neurologic monitoring (MMM).

**Methods:** We performed a retrospective analysis of pediatric TBI patients undergoing TCD integrated into MMM. Classic TCD characteristics included pulsatility indices and systolic, diastolic and mean flow velocities of the bilateral middle cerebral arteries. Model-based indices of cerebrovascular dynamics included the mean velocity index (Mx), compliance of the cerebrovascular bed (Ca), compliance of the cerebrospinal space (Ci), arterial time constant (TAU), critical closing pressure (CrCP) and diastolic closing margin (DCM). Classic TCD characteristics and model-based indices of cerebrovascular dynamics were investigated in relation to functional outcomes and intracranial pressure (ICP) using generalized estimating equations with repeated measures. Functional outcomes were assessed using the Glasgow Outcome Scale–Extended Pediatrics score (GOSE-Peds) at 12 months, post-injury.

**Results:** Seventy-two separate TCD studies were performed on twenty-five pediatric TBI patients. We identified that reduced Ci (estimate −5.986, *p* = 0.0309), increased CrCP (estimate 0.081, *p* < 0.0001) and reduced DCM (estimate −0.057, *p* = 0.0179) were associated with higher GOSE-Peds scores, suggestive of unfavorable outcome. We identified that increased CrCP (estimate 0.900, *p* < 0.001) and reduced DCM (estimate −0.549, *p* < 0.0001) were associated with increased ICP.

**Conclusion:** In an exploratory analysis of pediatric TBI patients, increased CrCP and reduced DCM and Ci are associated with unfavorable outcomes, and increased CrCP and reduced DCM are associated with increased ICP. Prospective work with larger cohorts is needed to further validate the clinical utility of these features.

## 1 Introduction

Traumatic brain injury (TBI) is a major cause of morbidity and mortality in children both across the United States as well as around the world (Hawley et al., 2002; Maas et al., 2017). Currently limited high-level evidence exists to guide clinical management, with existing level III evidence supporting maintenance of intracranial pressure (ICP) under 20 mmHg and cerebral perfusion pressure (CPP) above 40 mmHg (Kochanek et al., 2019). These thresholds are provided across a variety of pediatric age ranges and forms of trauma, making them likely insufficient for the optimal targeting that many children may need depending on such factors. Additional research is needed to understand whether other parameters of cerebral and systemic hemodynamics may be helpful toward optimizing neuroprotective strategies in pediatric TBI care.

Transcranial Doppler ultrasonography (TCD) and multimodality neurologic monitoring (MMM) have both emerged as techniques that may aid in clinical management of TBI patients by increasing insight into cerebral hemodynamics in a real-time basis and providing early warning of clinical deterioration as well as potential targets for therapeutic interventions (Appavu et al., 2019). Using TCD, flow velocities can be examined of the major basal cerebral arteries to better understand the nature of cerebral blood flow across different cerebrovascular territories (LaRovere et al., 2016). When TCD flow velocities are time-synchronized with MMM that includes ICP, arterial blood pressure (ABP) and electrocardiogram (ECG) data, a variety of unique model-based indices of cerebrovascular dynamics can be extracted (Varsos et al., 2014a). These include features that assess cerebrovascular pressure reactivity (CVPR), compliance of the cerebrovascular bed (Ca) and cerebrospinal space (Ci), arterial time constant (TAU), critical closing pressure (CrCP), and diastolic closing margin (DCM). While these TCD-derived features offer promise in individualizing care, there is limited literature to date examining such characteristics in pediatric TBI patients. Here, we aimed to investigate TCD-derived model-based indices of cerebrovascular dynamics in pediatric TBI patients as they relate to functional outcomes and ICP. We hypothesized that inefficient CVPR, decreased Ci, higher CrCP and lower DCM are associated with increased ICP and worsened functional outcomes.

## 2 Materials and methods

### 2.1 Study design

This is a retrospective cohort study from a prospectively collected clinical database of pediatric TBI patients admitted to Phoenix Children’s Hospital (Phoenix, Arizona, United States of America). The study was approved by the Institutional Review Board (No: 19–284).

### 2.2 Patients

Children ages 1–18 years of age with TBI who were hospitalized at the Phoenix Children’s Hospital Pediatric Intensive Care Unit from September 2014 to September October 2022 and underwent MMM that utilized intraparenchymal intracranial pressure (ICP) (Codman^®^, Integra Life Sciences^®^, Princeton, New Jersey, United States), invasive arterial blood pressure (ABP) and TCD were included. We excluded patients where ICP monitoring was only collected intermittently through an external ventricular drain (EVD) since it was not a continuous variable but included those who received both an EVD and intraparenchymal probe for ICP monitoring. Reasons for receiving ICP monitoring included Glasgow Coma Scales (GCS) scores ≤8 and GCS scores >8 with progressive neurologic decline, or patients who required sedative and/or paralytic therapies to address other comorbidities (e.g., splenic and/or liver lacerations). We did not include patients for whom TCD studies were performed exclusive of time-synchronized data integration into the MMM system. All patients were managed under standard of care and in accordance with an institutional protocol developed upon the most up-to-date pediatric TBI guidelines aimed toward maintaining ICP <20 mmHg and CPP >40 mmHg (Kochanek et al., 2012; Kochanek et al., 2019). To assess initial injury severity and determine the need for intracranial monitoring, GCS scores were made upon initial presentation to either the trauma bay or to the intensive care unit. Patient age, race, sex and TBI mechanisms were collected. TBI mechanisms were classified according to standardized pediatric TBI common data elements (Adelson et al., 2012).

### 2.3 Physiologic data collection

TCD was captured in the pediatric intensive care unit using M-Mode devices (Spencer Technologies^®^, Washington, United States or Novasignal Lucid^®^ systems, Los Angles, California, United States) of the middle cerebral arteries and integrated into multimodality neurologic monitoring hardware (Moberg CNS Monitor, Micromed^®^, Ambler PA) during the initial 7 days of admission while undergoing ICP monitoring. Model-based indices of cerebrovascular dynamics were calculated and processed using additional software (Intensive Care Monitor Plus [ICM+^®^], Cambridge, United Kingdom). These features included mean velocity index (Mx), Ca, Ci, TAU, CrCP, and DCM. A summary of the calculations of model-based indices of cerebrovascular dynamics is previously cited (Varsos et al., 2014a) and summarized in Table 1. Mx represents a moving Pearson correlation coefficient examining the relationship of TCD-derived mean flow velocities (MFVs) with ABP to investigate CVPR (Budohoski et al., 2012). Here, Mx values approaching 1 are representative of inefficient cerebrovascular pressure reactivity, whereas values approaching −1 are representative of efficient cerebrovascular pressure reactivity. Ca represents a similar moving Pearson correlation coefficient that examines features of TCD flow velocities that describe cerebral arterial blood volume (CABV) with the pulse amplitude of the ABP waveform (AMP_ABP_), whereas Ci is similarly calculated but instead compares CABV with the pulse amplitude of ICP waveform (AMP_ICP_) (Kim et al., 2009). TAU examines features of TCD flow velocities, ABP and CPP to examine the time for which cerebral blood flow stabilizes with a change in ABP (Czosnyka et al., 2012). CrCP is calculated from features of ICP, ABP, ECG, and TCD flow velocity waveforms to examine the theoretical ABP cutoff below which cerebral blood flow ceases (Varsos et al., 2013a). Diastolic closing margin (DCM) represents the difference between a patient’s diastolic blood pressure and CrCP and represents a reflection of cerebrovascular reserve (Varsos et al., 2014b). In addition to these TCD-derived model-based indices of cerebral dynamics, systolic flow velocities (SFV), mean flow velocities (MFV), diastolic flow velocities (DFV), and pulsatility indices (PIs) were also collected for each TCD test, in addition to the number of standard deviations from previously published normative values of critically ill sedated children (O’Brien, 2015). All TCD and MMM data collected for this analysis was screened for artifact by visual analysis. Artifactual data included non-sensical physiologic values, including negative values of ABP or ICP, and values of any physiologic data (e.g., ABP, ICP, TCD MFV, heart rate) without appropriate corresponding waveform morphologies visualized from multimodality neurologic monitoring data. Epochs that included such artifactual data was manually removed from data files before processing of model-based indices of cerebrovascular dynamics.

**TABLE 1:** Model based indices of cerebrovascular dynamics (Varsos et al., 2014a).

Term	Calculation	Units	Interpretation
Pulsatility Index (PI)	$\frac{{FV}_{systolic}-{FV}_{diastolic}}{{FV}_{mean}}$	-	
Mean Velocity Index (Mx)	Pearson correlation ( ${FV}_{mean}$ , ABP)	-	Degree of efficiency of cerebrovascular pressure reactivity in vascular bed insonated on TCD.
Cerebral Arterial Blood Volume (CaBV)	$∆CaBV\left(n\right)=Sa∙\overset{n}{\underset{i=1}{\sum }}\left({FV}_{a}\left(i\right)-meanFVa\right)∆t\left[{cm}^{3}\right]$	cm	Change in cerebral arterial blood volume
Compliance of the Cerebrovascular Bed (Ca)	$C_{a}=\frac{{AMP}_{CABV}}{{AMP}_{ABP}}$	$\frac{{cm}^{3}}{mmHg}$	Degree of compliance within vascular bed insonated on TCD.
Compliance of the Cerebrospinal Space (Ci)	$C_{i}=\frac{{AMP}_{CABV}}{{AMP}_{ICP}}$	$\frac{{cm}^{3}}{mmHg}$	Degree of compliance within cerebrospinal space of region insonated on TCD.
Arterial Time Constant (TAU)	$TAU=\frac{CPP∙{AMP}_{CABV}}{{FV∙AMP}_{ABP}}$	sec	Time in which arterial blood volume stabilizes after a change in ABP.
Critical Closing Pressure (CrCP)	$CCP=ABP-\frac{CPP}{\sqrt{{\left(TAU∙HR∙2\pi \right)}^{2}+1}}$	mmHg	The lower critical threshold of ABP below which small brain vessels collapse and CBF ceases
Diastolic Closing Margin (DCM)	${ABP}_{diastolic}-CCP$	mmHg	Differences between diastolic arterial blood pressure and critical closing pressure

Abbreviations: FV, flow velocity; ABP, arterial blood pressure; Sa, cross sectional area of the insonated vessel; cm, centimeters; TCD, transcranial Doppler ultrasound; Ca, compliance of the cerebrovascular bed; CABV, cerebral arterial blood volume; ABP, arterial blood pressure; mmHg, millimeters of mercury; TAU, arterial time constant; CPP, cerebral perfusion pressure; AMP, pulse amplitude; sec, seconds; HR, heart rate; CrCP, critical closing pressure; CBF, cerebral blood flow; DCM, diastolic closing margin.

### 2.4 Assessment

To investigate global functional outcomes, we measured Glasgow Outcome Scale Extended Pediatrics Revision (GOSE-Peds) scores during clinic follow-up 12 months after injury (Beers et al., 2012). GOSE-Peds scores ranged from 1 to 8, with lower scores indicating favorable outcome and higher scores representing unfavorable outcomes. As a secondary measure, we also examined intracranial pressure at the time during which each TCD study was performed. To explore whether differences in TCD features of cerebrovascular dynamics were associated with location of intracranial lesions, we collected the hemispheric location of subdural hematomas, epidural hematomas, contusions and intraparenchymal hemorrhages from initial computed tomography (CT) scans performed on each patient.

### 2.5 Statistical analysis

Continuous variables are expressed with median values and interquartile ranges [IQR], whereas categorical variables are expressed in counts and percentages. To examine the relationship of TCD-derived features with GOSE-Peds scores and ICP under a repeated measures model, we utilized generalized estimating equations to investigate the estimate and *p*-value for significance. Generalized estimation equations allows for modeling of data that is clustered, non-normative, longitudinal, and can be used to account for repeated measures (Carr and Chi, 1992). The estimate ascertained using generalized estimating equations represents the model coefficient, in which positive values represent positive associations and negative values represent negative associations. We performed the McNemar Chi-squared test to explore whether increased median values of any TCD-derived feature per patient were associated with the location of lesions on initial CT neuroimaging. Wilcoxon Rank-Sum test was used to explore differences in values of each TCD-derived feature within each middle cerebral artery (MCA) territory. Statistical analyses were performed using R Studio Version 3.4.1.

## 3 Results

### 3.1 Patient characteristics

Demographic and physiologic data is summarized in Table 2. Twenty-five patients were identified with TBI who underwent integrated MMM that included TCD with all patients having an initial GCS score ≤8, indicating that they all presented with severe TBI (Abdelmalik et al., 2019). Median age was 8.0 years (IQR: 3.5, 14.0). There were nineteen male (76.0%) and six female patients (24.0%). Eleven patients were Hispanic (44.0%), ten were Caucasian (40.0%), two were Native American (8.0%), one was Asian American (4.0%) and one was African American (4.0%). In this patient cohort, nine patients (36.0%) experienced closed head injury, nine patients (36.0%) experienced penetrating head injury, six patients (24.0%) experienced abusive head trauma, one patient (4.0%) experienced crush head injury, and no patients (0.0%) experienced blast head injury. Two patients (8.0%) had initial neuroimaging findings demonstrating symmetric bifrontal contusions, seven (28.0%) had left hemispheric subdural hematomas, four (16.0%) had left hemispheric intraparenchymal hemorrhages, one (4.0%) had a left hemispheric epidural hematoma, five (20.0%) had right hemispheric subdural hematomas, five (20.0%) had right hemispheric contusions, and one (4.0%) had extensive right hemispheric cerebral edema. No patients were identified on anatomical neuroimaging to have experienced post-traumatic cerebrovascular vasospasms. Seventy-two TCD studies were performed in total with MCA insonation among the twenty-five patients in this cohort, including twenty-nine studies (40.3%) where both the left and right MCA territories were insonated at the same time, sixteen studies that were performed studies exclusive to the left MCA territory (22.2%) and twenty-two studies (30.6%) that were performed exclusive to the right MCA territory. Each patient received between one to eleven TCDs per admission with a median of 2.0 procedures per patient (IQR: 2.0, 3.0), all during the initial 7 days of monitoring in the intensive care unit. Twenty-four patients (96.0%) had up to one TCD performed a day whereas one patient (4.0%) underwent twice-daily TCDs for 4 days and once-daily TCDs for 3 days. Median length of TCD recordings was 50.67 min (IQR: 32.25, 81.08). Within our cohort, we identified significantly higher values in the left MCA territory for Mx (*p* = 0.0260), Ca (*p* = 0.0005) and Ci (*p* = 0.0019) as compared to right MCA territory (Supplementary Table S3).

**TABLE 2:** Patient characteristics.

Characteristics (*n* = 75)	Number (%) of patients
Female, number	6 (24.0)
Hispanic	11 (44.0)
Caucasian	10 (40.0)
Native American	2 (8.0)
Asian American	1 (1.0)
Closed Head Injury	9 (36.0)
Penetrating Head Injury	9 (36.0)
Crush Head Injury	1 (4.0)
Blast Head Injury	0 (0.0)
Abusive Head trauma	6 (24.0)
Bitemporal TCD insonation	29 (40.3)
Left MCA insonation	16 (22.3)
Right MCA insonation	22 (30.6)
	Median (IQR)
Age	11.0 (3.5, 14.0)
Glasgow Coma Scale, at presentation	4.0 (3.5, 5.0)
GOSE-Peds, 12 months post-injury	4.0 (2.5, 5.0)
Intracranial Pressure, mmHg	15.35 (10.20, 17.43)
TCD performed per patient, n	2.0 (1.0, 3.0)
Length of TCD recordings, minutes	50.67 (32.25, 81.08)
Mean flow velocities, left (cm/sec)	65.08 (50.92, 90.33)
Mean flow velocities, left (standard deviations from normative values for age)	0.52 (−0.71, 2.08)
Mean flow velocities, right (cm/sec)	69.61 (45.28, 81.41)
Mean flow velocities, left (standard deviations from normative values for age)	0.32 (−0.81, 1.06)
Systolic flow velocities, left (cm/sec)	115.50 (87.12, 136.15)
Systolic flow velocities, left (standard deviations from normative values for age)	0.39 (−0.91, 1.34)
Systolic flow velocities, right (cm/sec)	99.15 (78.78, 117.50)
Systolic flow velocities, right (standard deviations from normative values for age)	−0.47 (−1.16, 0.39)
Diastolic flow velocities, left (cm/sec)	42.4 (33.7, 50.8)
Diastolic flow velocities, left (standard deviations from normative values for age)	0.16 (−0.36, 0.94)
Diastolic flow velocities, right (cm/sec)	39.6 (28.9, 50.0)
Diastolic flow velocities, right (standard deviations from normative values for age)	0.07 (−0.79, 0.79)
Pulsatility indices, left	1.02 (0.85, 1.06)
Pulsatility indices, right	1.04 (0.84, 1.23)
Pulsatility indices (standard deviation from normative values for age)	−0.22 (−1.09, 0.83)
Mean velocity index (Mx), left	0.30 (0.07, 0.50)
Mean velocity index (Mx), right	0.07 (−0.04, 0.30)
Compliance of the cerebrovascular bed (Ca), left	0.0003 (0.0002, 0.0004)
Compliance of cerebrovascular bed (Ca), right	0.0002 (0.0001, 0.0001)
Compliance of cerebrospinal space (Ci), left	0.1118 (0.0025, 1.6970)
Compliance of the cerebrospinal space (Ci), right	0.0028 (0.0016, 0.0094)
Arterial time constant (TAU), left MCA (seconds)	0.0003 (0.0002, 0.0005)
Arterial time constant (TAU), right MCA (seconds)	0.0002 (0.0002, 0.0003)
Critical closing pressure (CrCP), left MCA (mmHg)	17.14 (12.08, 24.70)
Critical closing pressure (CrCP), right (mmHg)	15.24 (11.47, 20.35)
Diastolic closing margin (DCM), left (mmHg)	35.84 (32.46, 42.63)
Diastolic closing margin (DCM), right (mmHg)	40.83 (35.34, 47.63)

Abbreviations: n, count; %, percentage; TCD, transcranial Doppler ultrasound; MCA, middle cerebral artery; TCD, transcranial Doppler ultrasound; n, count; IQR, interquartile range; GOSE-Peds, Glasgow Outcome Scale Extended–Pediatrics; cm, centimeters; sec, seconds; Mx, mean velocity index; Ca, compliance of the cerebrovascular bed; Ci, compliance of the cerebrospinal space; TAU, arterial time constant; CrCP, critical closing pressure; DCM, diastolic closing margin; mmHg, millimeters of mercury.

### 3.2 Association with functional outcomes

The association of TCD characteristics with GOSE-Peds scores is summarized in Table 3 and Supplementary Table S1. When assessing TCD characteristics over both MCA territories, we identified that reduced Ci (estimate −5.986, *p* = 0.0309), increased CrCP (estimate 0.081, *p* < 0.0001), and reduced DCM (estimate −0.057, *p* = 0.0179) were associated with higher GOSE-Peds scores, suggestive of unfavorable outcome. When assessing both MCA territories, we did not observe that SFV, MFV, DFV, Mx, Ca, or TAU were associated with GOSE-Peds scores. When specifically assessing each MCA territory, we observed that higher GOSE-Peds scores were associated with increased Mx values over the left MCA territory (estimate 2.022, *p* = 0.0023), reduced Ci values over both the left MCA territory (estimate 18.19, *p* = 0.0016) and right MCA territory (estimate −3.63, *p* = 0.0133), increased CrCP over the right MCA territory (estimate 0.080, *p* < 0.0001), and reduced DCM over the right MCA territory (estimate −0.075, *p* < 0.0001).

**TABLE 3:** Association of TCD characteristics with GOSE-Peds scores, 12-month post-injury.

	Estimate	Model SE	Robust SE	*p*-value
Mean flow velocities	−0.005	0.009	0.012	0.6766
Mean flow velocities, standard deviations from normative values	−0.031	0.128	0.135	0.8169
Systolic flow velocities, absolute	−0.002	0.008	0.010	0.8391
Systolic flow velocities, standard deviations from normative values	−0.265	0.171	0.277	0.3391
Diastolic flow velocities	−0.006	0.0147	0.020	0.7584
Diastolic flow velocities, standard deviations from normative values	0.076	0.169	0.244	0.7570
Pulsatility index (PI), absolute	0.293	0.907	0.855	0.7315
Pulsatility index (PI), standard deviations from normative values	0.0039	0.159	0.159	0.9803
Mean velocity index (Mx)	0.9276	0.573	0.594	0.1186
Compliance of the cerebrovascular bed (Ca)	−21.470	126.500	111.200	0.8468
**Compliance of the cerebrospinal space (Ci)**	**−5.986**	**−18.190**	**5.749**	**0.0309**
Arterial time constant (TAU)	−77.770	127.700	124.800	0.5332
**Critical closing pressure (CrCP)**	**0.081**	**0.016**	**0.020**	**<0.0001**
**Diastolic closing Margin (DCM)**	**−0.057**	**0.020**	**0.024**	**0.0179**

Abbreviations: TCD, transcranial Doppler ultrasound; GOSE-Peds, Glasgow Outcome Scale Extended–Pediatrics; MFV, mean flow velocity; SFV, systolic flow velocity; DFV, diastolic flow velocity; PI, pulsatility index; Mx, mean velocity index; Ca, compliance of the cerebrovascular bed; Ci, compliance of the cerebrospinal space; TAU, arterial time constant; CrCP, critical closing pressure; DCM, diastolic closing margin; SE, standard error.

### 3.3 Association of intracranial pressure

The association of TCD characteristics with ICP is summarized in Table 4 and Supplementary Table S2. When assessing TCD characteristics over both MCA territories, we observed that increased CrCP (estimate 0.900, *p* < 0.0001) and reduced DCM (estimate −0.549, *p* = 0.0158) were associated with increased ICP. We did not observe that FV, Mx, Ca, Ci, or TAU were associated with ICP. When specifically assessing each MCA territory, we observed that increased ICP was associated with reduced Ci over the left MCA territory (estimate −2.517, *p* = 0.0336), increased CrCP over the left MCA territory (estimate 0.667, *p* = 0.0005) and right MCA territory (estimate 1.002, *p* < 0.0001), and reduced DCM over the left MCA territory (estimate −0.445, *p* = 0.0079) and right MCA territory (estimate −0.747, *p* = 0.0020).

**TABLE 4:** Association of TCD characteristics with intracranial pressure.

	Estimate	Model SE	Robust SE	*p*-value
Mean flow velocities	−0.025	0.013	0.012	0.6244
Mean flow velocities, standard deviations from normative values	−0.973	0.670	0.732	0.1839
Systolic flow velocities	−0.001	0.028	0.029	0.9746
Systolic flow velocities, standard deviations from normative values	−1.130	0.704	0.839	0.1779
Diastolic flow velocities	0.083	0.072	0.092	0.3684
Diastolic flow velocities, standard deviations from normative values	−1.372	0.757	0.752	0.0682
Pulsatility index (PI)	1.463	4.789	3.224	0.6500
Pulsatility index (PI), standard deviations from normative values	0.130	0.509	0.666	0.8447
Mean velocity index (Mx)	2.905	2.597	2.384	0.2011
Compliance of the cerebrovascular bed (Ca)	257.600	321.00	150.700	0.0775
Compliance of the cerebrospinal space (Ci)	−1.200	0.825	0.897	0.2120
Arterial time constant (TAU)	83.3600	181.400	51.440$cr	0.1272
**Critical closing pressure (CrCP)**	**0.900**	**0.0318**	**0.0847**	**<0.0001**
**Diastolic closing Margin (DCM)**	**−0.549**	**0.088**	**0.245**	**0.0158**

Abbreviations: TCD, transcranial Doppler ultrasound; MFV, mean flow velocity; SFV, systolic flow velocity; DFV, diastolic flow velocity; PI, pulsatility index; Mx, mean velocity index; Ca, compliance of the cerebrovascular bed; Ci, compliance of the cerebrospinal space; TAU, arterial time constant; CrCP, critical closing pressure; DCM, diastolic closing margin; SE, standard error; MFV.

### 3.4 Association with lesions

In our exploratory analysis of lesion location with TCD characteristics, we did not identify that elevated values for any TCD characteristic were associated with the location of intracranial lesions (Supplementary Table S4).

## 4 Discussion

In this retrospective cohort study, we identified that after pediatric TBI, reduced Ci, increased CrCP and reduced DCM are associated with worsened functional outcomes. We also identified that increased CrCP and reduced DCM were associated with increased ICP. To our knowledge, this is the first study to investigate TCD-derived model-based indices of cerebrovascular dynamics in pediatric TBI patients.

A key concept of neuroprotection in pediatric TBI care is maintenance of appropriate cerebral perfusion to allow for adequate brain tissue oxygenation and cortical function (Appavu et al., 2019). Given the widespread adoption of ICP monitoring, cerebral perfusion pressure (CPP) monitoring has emerged as a mainstay method of assessing for adequate cerebral perfusion. CPP monitoring, however, does not directly assess cerebral perfusion but rather is a calculated derivative negating intracranial pressure from systemic ABP. While CPP monitoring is practical and CPP thresholds are linked with functional outcomes, it may not always be clear as to whether a change in CPP represents a corresponding response in cerebral perfusion. For instance, a rise in arterial carbon dioxide content may lead to an increase in cerebral blood flow and ICP, but if ABP stays stable or reduces as an appropriate response of efficient CVPR, the calculated CPP number will fall (Supplementary Figure S1). Due to such circumstances, better methods of monitoring cerebral blood flow may be insightful in effectively managing patients toward optimal cerebral perfusion.

As a closer measure of cerebral perfusion than CPP itself, TCD flow velocity characteristics have been studied in pediatric TBI patients. Several prospective observational studies have associated either elevated PIs or low EDV with increased ICP or unfavorable outcomes (Vavilala et al., 2007; Figaji et al., 2009; Melo et al., 2011). A few studies have evaluated dynamic testing of CVPR or carbon dioxide reactivity, demonstrating that inefficient CVPR or limited carbon dioxide reactivity are associated with unfavorable outcomes (Maa et al., 2016; Hanalioglu et al., 2022). Low flow velocities combined with increased pulsatility indices have been linked toward increased ICP. Caution toward such interpretation is needed, as reductions in ABP can also result in reduced flow velocities and increased pulsatility indices. The ability to synchronize TCD FVs with ABP, ICP and ECG characteristics allow for such factors to be considered, and from this several model-based indices of cerebrovascular dynamics now exist. Elevated CrCP represents an increased requirement for sufficient systemic blood pressure support to provide adequate cerebral blood flow, and a reduce diastolic closing margin represents diminished cerebrovascular reserve toward adequate cerebral perfusion. Animal models have provided validation to support the notion that reduction of diastolic ABP below CrCP is associated with cessation of diastolic cerebral blood flow (Varsos et al., 2014b), and clinical studies of adult TBI patients have demonstrated that increases in CrCP can be observed during ICP plateau waves (Varsos et al., 2013b). CrCP and DCM may represent features that better characterize cerebral perfusion than calculated CPP values and these features may offer improved an improved therapeutic target to optimize cerebral perfusion. Our observation that reduced Ci is associated with unfavorable outcomes is unique and suggests that compliance of the cerebrospinal space may be the most important component of the intracranial vault as it relates to functional outcomes. While we know that high ICP can be associated with unfavorable outcomes and increased risk of cerebral herniation, increases in ICP can reflect change in any component of the intracranial vault including the cerebrovascular bed or cerebrospinal space. We often worry about cerebral edema or obstructive hydrocephalus as secondary insults that are reflected in high ICP, and these pathogenic processes may be better reflected by reductions in Ci, whereas increases in ICP due to increased cerebral blood flow may be reflected by increases in Ca. While a variety of therapeutic strategies exist to mitigate against intracranial hypertension, each of these techniques may have limited efficacy depending on the underlying source of intracranial hypertension. Techniques such as hypertonic saline may be most efficient when cerebral edema is the culprit, whereas obstructive hydrocephalus may be more efficiently treated with placement of an EVD, and hyperemic cerebral blood flow may be best addressed with optimized ventilation. Intravascular and extravascular sources of intracranial hypertension may have differentiating influences on Ca and Ci (Varsos et al., 2014a), and trends of such characteristics, combined with CrCP and DCM, may allow for a better understanding of which therapeutic strategy may be most effective toward mitigation of intracranial hypertension for a given patient.

We performed an exploratory subset analysis of both left and right MCA territories given the added advantage that TCD has in evaluating multiple different cerebrovascular territories. We observed elevated values for Mx, Ca, and Ci over the left MCA territory as compared to the right, but due to our low sample size we are unable to ascertain whether these findings our generizable or more reflective of our single-center cohort. With that in mind, we find it noteworthy that Mx was associated with outcomes specifically over the left MCA region, raising the question as to whether optimizing cerebrovascular pressure reactivity may be more relevant over territories representing eloquent cortex (i.e., language cortices). Our observation that increased CrCP and reduced DCM are associated with increased ICP is unsurprising, given that ICP characteristics are factored into the calculation of each of these indices of cerebrovascular dynamics. We note that overall Mx values were not associated with functional outcomes, which contrasts findings observed with Mx in adult TBI patients (Czosnyka et al., 1999) as well as other CVPR biomarkers identified in pediatric TBI patients (Brady et al., 2009; Lewis et al., 2015; Appavu et al., 2021). We note that most of the patients within this cohort were also part of a larger cohort in which we previously demonstrated that elevated PRx values were associated with unfavorable outcomes, in addition to other ICP-derived model-based indices of CVPR (Appavu et al., 2021). It remains unclear why we have observed a lack of association to outcome with Mx in opposition to PRx, and we speculate that our sample size may be underpowered to assess such an outcome. With regards to PRx, ICP monitoring is often placed at the side of the lesion, and investigations of CVPR specific to the region of injury may be better associated with functional outcomes, although we did not observe that higher Mx values were associated with the region of intracranial lesions. It is also possible that while PRx and Mx both evaluate CVPR, PRx may offer a better assessment of injury severity whereas Mx may allow for a more precise assessment of regional CVPR. As part of our larger pediatric TBI cohort, we have previously described that we did not identify age or initial GCS as biomarkers predictive of functional outcomes, and we did observe that the dose of intracranial hypertension was associated with unfavorable outcomes (Appavu et al., 2021).

Our study was exploratory and limited by a retrospective study design, single-site data collection and a small sample size. Our study population appeared skewed toward an increased population of males and Caucasian and Hispanic individuals. Because of our low sample size, we can explore directional associations of these features with functional outcomes and intracranial pressure, but we are not able to establish cutoffs for which there is increased risk. We focused our study on TCD evaluations of the middle cerebral arteries, and it may be of value to investigate these parameters in relation to basilar artery, posterior cerebral artery, and anterior cerebral artery vasculature, as these regions may represent territories of circulation that supply cortical or brainstem regions that are needed for the necessary function for favorable outcomes. Future larger, prospective studies with adequate sample size and normally distributed patient populations will be necessary for increasing the validity of these features as well as understanding if they are generizable (Cabella et al., 2017).

## 5 Conclusion

In this exploratory analysis of pediatric TBI patients, increased CrCP and reduced DCM and Ci are associated with unfavorable outcomes, and increased CrCP and reduced DCM are associated with increased ICP. Prospective work is needed to determine whether management strategies that decrease CrCP and increase DCM and Ci may optimize cerebral hemodynamics and improve functional outcomes.

## Data Availability

The raw data supporting the conclusions of this article will be made available by the authors, without undue reservation.

## Supplementary material

Supplementary Table S1 Association of TCD characteristics with GOSE-Peds scores, 12-months post-injury, based on left or right MCA insonation.

Supplementary Table S2 Association of TCD characteristics with intracranial pressure, based on left or right MCA insonation.

Supplementary Table S3 Differences in neurosonologic features based on hemisphere of insonation.

Supplementary Table S4 Association of elevated TCD characteristic with location of intracranial lesions.
