# Supplementary material for: Cerebrovascular dynamics after pediatric traumatic brain injury
Source: Front Physiol. 2023 Feb 17;14:1093330. doi: 10.3389/fphys.2023.1093330 (PMC9981944; doi:10.3389/fphys.2023.1093330)
Supplement: Supplementary file 1 [file DataSheet1.pdf]

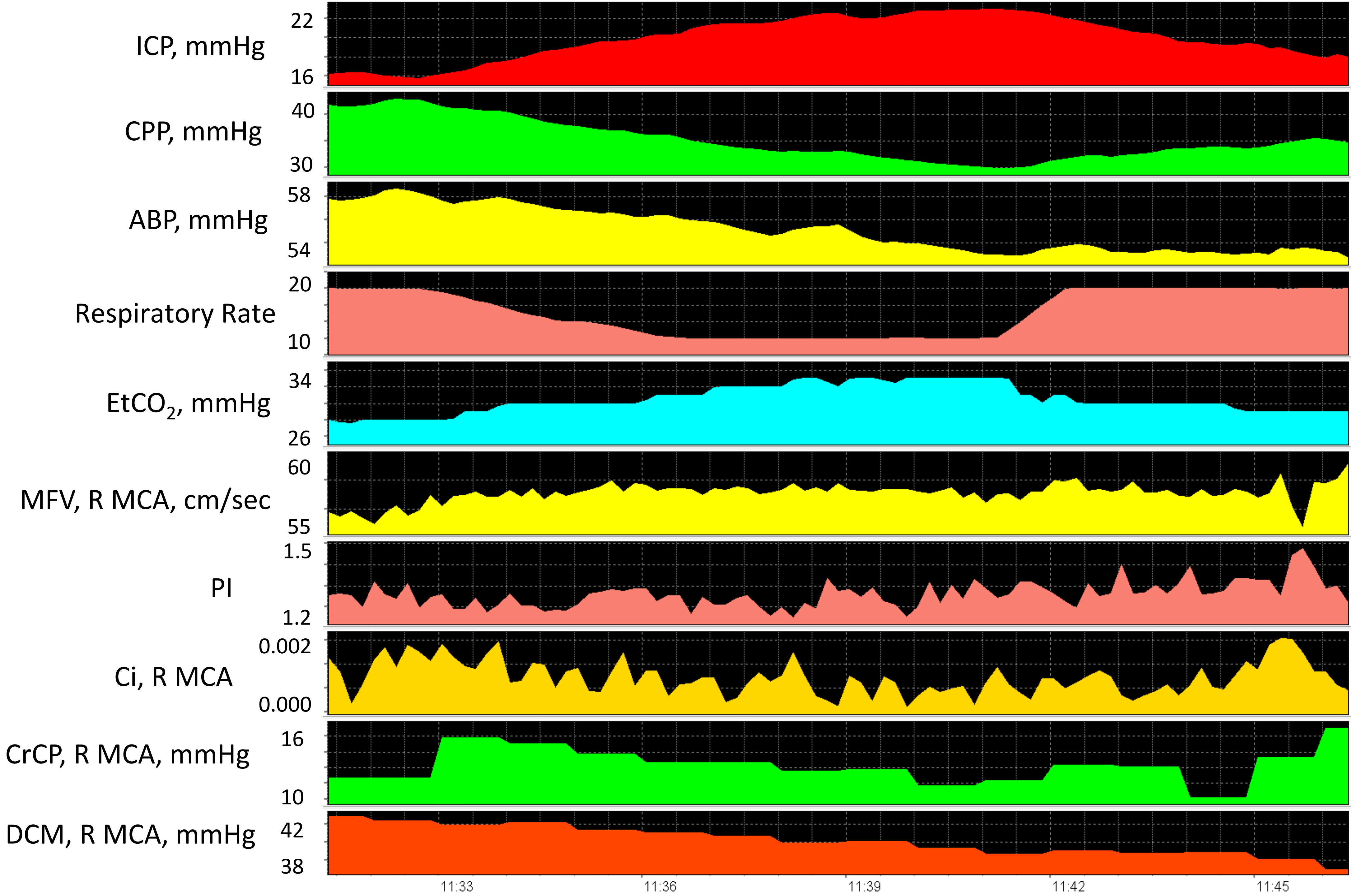
ICP, mmHg
22
16
CPP, mmHg
40
30
ABP, mmHg
58
54
Respiratory Rate
20
10
EtCO2, mmHg
34
26
MFV, R MCA, cm/sec
60
55
PI
1.5
1.2
Ci, R MCA
0.002
0.000
CrCP, R MCA, mmHg
16
10
DCM, R MCA, mmHg
42
38
11:33
11:36
11:39
11:42
11:45

Supplemental Figure 1: Example of an 8-year-old female with severe traumatic brain injury who undergoes a TCD study of the right MCA territory with discordant findings from TCD flow velocities and CPP. A reduction in respiratory rate from 20 to 12 leads to an increase in right MCA MFVs from 55 to 58 cm/sec, with a corresponding rise in ICP from 16 to 22 mmHg, a rise in $EtCO_2$ from 28 to 34 mmHg and a drop in ABP from 58 to 55 mmHg. Despite an increase in right MCA MFV, the calculated CPP value drops from 42 to 32 mmHg. The rise in ICP is also associated with an initial rise in CrCP and reduction in DCM. The initial rise in CrCP is offset with a reduction in ABP. The reduction of arterial blood pressure with a concordant rise in ICP and $EtCO_2$ may reflect a mechanism of efficient cerebrovascular pressure reactivity to regulate cerebral blood flow as a response to a change in ventilation. Abbreviations: TCD, transcranial Doppler ultrasound; MCA, middle cerebral artery; ICP, intracranial pressure; CPP, cerebral perfusion pressure; ABP, arterial blood pressure; $EtCO_2$, end-tidal carbon dioxide; mmHg, millimeters of mercury; R, right; MFV, mean flow velocities; cm, centimeters; sec, seconds; PI, pulsatility index; Ci, compliance of the cerebrospinal space; CrCP, critical closing pressure; DCM, diastolic closing margin.

Supplemental Table 1: Association of TCD characteristics with GOSE-Peds scores, 12-months post-injury, based on left or right MCA insonation

	Left MCA				Right MCA			
	Estimate	Model SE	Robust SE	p-value	Estimate	Model SE	Robust SE	p-value
Mean flow velocities	-0.004	0.013	0.012	0.7578	-0.004	0.013	0.018	0.8832
Mean flow velocities, standard deviations from normative values	0.013	0.145	0.111	0.9059	-0.078	0.212	0.286	0.7860
Systolic flow velocities, absolute	-0.003	0.011	0.010	0.7718	0.004	0.011	0.015	0.7836
Systolic flow velocities, standard deviations from normative values	-0.004	0.013	0.018	0.8332	-0.152	0.246	0.421	0.717
Diastolic flow velocities, absolute	-0.017	0.022	0.018	0.3222	0.010	0.0161	0.018	0.6702
Diastolic flow velocities, standard deviations from normative values	-0.262	0.255	0.229	0.2515	0.249	0.235	0.278	0.3696
Pulsatility index (PI), absolute	0.591	1.396	0.807	0.4638	-0.093	1.241	1.124	0.9342
Pulsatility index (PI), standard deviations from	-0.055	0.240	0.200	0.7850	-0.092	0.221	0.185	0.5993

normative values								
**Mean velocity index (Mx)**	**2.022**	**0.733**	**0.664**	**0.0023**	0.787	0.870	1.063	0.4592
Compliance of the cerebrovascular bed (Ca)	-214.900	148.600	197.700	0.2771	-99.500	1680.00	1710.00	0.9536
**Compliance of the cerebrospinal space (Ci)**	**-18.190**	**4.228**	**5.749**	**0.0016**	**-3.628**	**4.640**	**1.453**	**0.0133**
Arterial time constant (TAU)	-163.600	112.700	137.400	0.2337	48.740	940.200	767.500	0.9494
**Critical closing pressure (CrCP)**	0.030	0.063	0.046	0.5192	**0.080**	**0.017**	**0.019**	**<0.0001**
**Diastolic closing margin**	-0.131	0.062	0.089	0.1444	**-0.075**	**0.023**	**0.019**	**<0.0001**

Abbreviations: TCD, transcranial Doppler ultrasound; GOSE-Peds, Glasgow Outcome Scale Extended – Pediatrics; MCA, middle cerebral artery; MFV, mean flow velocity; SFV, systolic flow velocity; DFV, diastolic flow velocity; PI, pulsatility index; Mx, mean velocity index; Ca, compliance of the cerebrovascular bed; Ci, compliance of the cerebrospinal space; TAU, arterial time constant; CrCP, critical closing pressure; DCM, diastolic closing margin; SE, standard error; MFV, mean flow velocity; SFV, systolic flow velocity; DFV, diastolic flow velocity.

Supplemental Table 2: Association of TCD characteristics with intracranial pressure, based on left or right MCA insonation

	Left MCA				Right MCA			
	Estimate	Model SE	Robust SE	p-value	Estimate	Model SE	Robust SE	p-value
Mean flow velocities	-0.070	0.042	0.040	0.0853	-0.055	0.066	0.051	0.2858
Mean flow velocities, standard deviations from normative values	-0.509	0.561	0.658	0.4391	-1.529	1.593	1.536	0.3196
Systolic flow velocities	-0.029	0.028	0.031	0.3618	-0.018	0.046	0.032	0.5843
Systolic flow velocities, standard deviations from normative values	-1.023	0.703	0.837	0.2216	-1.269	0.718	0.845	0.1330
**Diastolic flow velocities**	**-0.1347**	**0.061**	**0.062**	**0.0305**	-0.200	0.137	0.108	0.0630
Diastolic flow velocities, standard deviations from normative values	0.8254	1.364	0.248	0.5085	-1.403	0.754	0.914	0.1245
**Pulsatility index (PI)**	-0.473	4.274	3.086	0.8781	**-0.008**	**0.002**	**0.003**	**0.0060**
Pulsatility index (PI), standard deviations from	-0.68	0.797	0.719	0.3441	0.4876	0.674	0.875	0.5775

normative values								
Mean velocity index (Mx)	4.274	3.292	3.209	0.1829	4.594	3.034	2.704	0.0893
Compliance of the cerebrovascular bed (Ca)	184.700	245.300	1.260	0.2077	2544.00	10930.000	5703.000	0.6566
**Compliance of the cerebrospinal space (Ci)**	**-2.517**	**0.967**	**1.185**	**0.0336**	-0.779	1.265	0.814	0.3385
Arterial time constant (TAU)	119.000	149.900	84.190	0.1573	4702.000	6654.000	3754.000	0.2104
**Critical closing pressure (CrCP)**	**0.667**	**0.079**	**0.193**	**0.0005**	**1.002**	**0.006**	**0.004**	**<0.0001**
**Diastolic closing margin**	**-0.445**	**0.123**	**0.168**	**0.0079**	**-0.747**	**0.092**	**0.242**	**0.0020**

Abbreviations: TCD, transcranial Doppler ultrasound; MCA, middle cerebral artery; MFV, mean flow velocity; SFV, systolic flow velocity; DFV, diastolic flow velocity; PI, pulsatility index; Mx, mean velocity index; Ca, compliance of the cerebrovascular bed; Ci, compliance of the cerebrospinal space; TAU, arterial time constant; CrCP, critical closing pressure; DCM, diastolic closing margin; SE, standard error.

Supplemental Table 3: Differences in neurosonologic features based on hemisphere of insonation.

Neurosonologic feature	p-value
Mean flow velocities, cm/sec	0.7978
Mean flow velocities, standard deviation from normative values	0.5002
Systolic flow velocities, cm/sec	0.1202
Systolic flow velocities, standard deviation from normative values	0.0733
Diastolic flow velocities, cm/sec	0.2093
Diastolic flow velocities, standard deviation from normative values	0.3500
Pulsatility indices (PI)	0.9714
Pulsatility indices (PI), standard deviation from normative values	0.9224
**Mean velocity index (Mx)**	**0.0260**
**Compliance of the cerebrovascular bed (Ca)**	**0.0005**
**Compliance of the cerebrospinal space (Ci)**	**0.0019**
Arterial time constant (TAU)	0.1704
Critical closing pressure (CrCP)	0.2109
Diastolic closing margin (DCM)	0.1283

Test performed using the Wilcoxon Rank-Sum test. Abbreviations: PI, pulsatility index; Mx, mean velocity index; Ca, compliance of the cerebrovascular bed; Ci, compliance of the cerebrospinal space; TAU, arterial time constant; CrCP, critical closing pressure; DCM, diastolic closing margin; cm, centimeters; sec, seconds.

Supplemental Table 4: Association of elevated TCD characteristic with location of intracranial lesions.

TCD Characteristic	Chi-squared	Df	p-value
Mean flow velocities, cm/sec	0.00	1.00	1.000
Systolic flow velocities, cm/sec	0.00	1.00	1.000
Diastolic flow velocities, cm/sec	0.00	1.00	1.000
Pulsatility indices (PI)	0.57	1.00	0.4497
Mean velocity index (Mx	2.25	1.00	0.1336
Compliance of the cerebrovascular bed (Ca)	0.00	1.00	1.0000
Compliance of the cerebrospinal space (Ci)	0.80	1.00	0.3711
Arterial time constant (TAU)	0.00	1.00	1.000
Critical closing pressure (CrCP)	0.00	1.00	1.000
Diastolic closing margin (DCM)	0.00	1.00	1.000

Test performed using the McNemar Chi-Squared test. Abbreviations: PI, pulsatility index; Mx, mean velocity index; Ca, compliance of the cerebrovascular bed; Ci, compliance of the cerebrospinal space; TAU, arterial time constant; CrCP, critical closing pressure; DCM, diastolic closing margin; cm, centimeters; sec, seconds.
